# Prehensile and Non-Prehensile Robotic Pick-and-Place of Objects in Clutter Using Deep Reinforcement Learning

**DOI:** 10.3390/s23031513

**Published:** 2023-01-29

**Authors:** Muhammad Babar Imtiaz, Yuansong Qiao, Brian Lee

**Affiliations:** Software Research Institute, Technological University of the Shannon: Midland Midwest, N37 HD68 Athlone, Ireland

**Keywords:** prehensile, non-prehensile, robotic manipulation, Markov decision process, deep reinforcement learning, deep Q-network, fully convolutional network, DenseNet-121

## Abstract

In this study, we develop a framework for an intelligent and self-supervised industrial pick-and-place operation for cluttered environments. Our target is to have the agent learn to perform prehensile and non-prehensile robotic manipulations to improve the efficiency and throughput of the pick-and-place task. To achieve this target, we specify the problem as a Markov decision process (MDP) and deploy a deep reinforcement learning (RL) temporal difference model-free algorithm known as the deep Q-network (DQN). We consider three actions in our MDP; one is ‘grasping’ from the prehensile manipulation category and the other two are ‘left-slide’ and ‘right-slide’ from the non-prehensile manipulation category. Our DQN is composed of three fully convolutional networks (FCN) based on the memory-efficient architecture of DenseNet-121 which are trained together without causing any bottleneck situations. Each FCN corresponds to each discrete action and outputs a pixel-wise map of affordances for the relevant action. Rewards are allocated after every forward pass and backpropagation is carried out for weight tuning in the corresponding FCN. In this manner, non-prehensile manipulations are learnt which can, in turn, lead to possible successful prehensile manipulations in the near future and vice versa, thus increasing the efficiency and throughput of the pick-and-place task. The Results section shows performance comparisons of our approach to a baseline deep learning approach and a ResNet architecture-based approach, along with very promising test results at varying clutter densities across a range of complex scenario test cases.

## 1. Introduction

The term Industry 4.0 has been around for more than a decade now. As the fourth industrial revolution was all about automation, industry became familiar with various cutting-edge technologies that were crucial to achieving the goal of automation, such as digital twins, cyber–physical systems, smart manufacturing, Internet of Things (IoT), etc.

The deployment of robotics in the industry, in one way or another, can be traced back almost 50 years. Usually, robotic manipulations can be divided into two kinds: prehensile and non-prehensile manipulation. Prehensile motion [1] involves grasping or capturing, whereas non-prehensile motion [2] is where propelling or pushing is involved instead of grasping. An example of prehensile and non-prehensile motion is shown in Figure 1 from [3]. Well-learned coordination between these two robotic manipulations can lead to higher success rates of pick-and-place; for instance, non-prehensile manipulation can create space between tightly packed objects, thus making grasping possible.

On the other hand, prehensile manipulation will pick objects and facilitate collision-free and more accurate pushing. A substantial amount of work has been conducted on prehensile and non-prehensile robotic manipulations in the past, but mostly, they have been researched independently of each other [4]. There is still a considerable research gap and potential for exploration in the area of the combination of these robotic manipulations. Non-prehensile manipulation has been primarily used to accurately deal with the poses of the objects [2]. In a combined approach involving grasping and pushing, pushing goals are quite flexible; for example, they can create space between objects, change the pose of objects, and crack clutter [5]. This flexibility factor was not addressed in different model-based [6,7,8] and data-driven [9,10,11] approaches in the past. Model-based approaches are those which are based on a static model, whereas data-driven approaches are those which involve any kind of machine learning through data. In the area of prehensile manipulation, there have been different approaches to learning successful grasping policies, some based on optimizing affordances by learning from previous experiences [12,13,14] and some learnt through grasp stability measures [15,16,17]. These approaches do not address the planning or learning of sequences of prehensile and non-prehensile manipulation actions to maximize the success rate of grasping and pushing. There are some hard-coded techniques that are dependent on domain knowledge for implementing single-action push–grasp but, obviously, they have very limited scope [2].

In this study, we present a framework in which the agent learns to perform a sequence of prehensile and non-prehensile robotic manipulations through model-free temporal difference off-policy deep reinforcement learning (Q-learning). This approach does not require any kind of prior domain knowledge or geometrical pose information like some previous approaches, which will be further discussed in the next section. The major objectives achieved in this study are as follows:

- We designed a self-supervised deep reinforcement learning-based data-driven framework. We deploy a memory-efficient joint network in this framework, unlike the approaches in the past. This framework successfully enables the agents to learn and perform a sequence of prehensile and non-prehensile operations together on regular- and irregular-shaped objects without causing any bottleneck situations, unlike preceding work. This learning is achieved through trial and error.
- The sliding or pushing operations are learnt in a bidirectional manner (left and right), which then further enables grasping operation. This learning of bidirectional non-prehensile manipulation directly elevates the progress of unidirectional non-prehensile manipulation. This contrasts with approaches in the past using hard-coded techniques, domain-based knowledge such as heuristics, or unidirectional prehensile manipulations [2].
- Our agents learn and converge to the optimal policies through a joint framework of end-to-end memory-efficient deep networks. In end-to-end networks, the model learns all the steps between the initial input phase and the final output result; therefore, memory management is essential to avoid bottleneck situations while using minimal resources. This joint framework takes the visual state of the workspace as an input, and in return, outputs the Q-values depicting the expected return for three different potential actions: grasping, sliding left, and sliding right. Once the Q-values are received, the action associated with the highest Q-value is selected as it predicts the maximum expected future reward. This whole procedure will be explained in detail in Section 3 but even this brief overview clearly shows that our approach contrasts with the approaches of the past, where each and every object was considered individually and hand-designed features and heuristics were crucial for the action selection part [18].

In light of the abovementioned research objectives of this paper, the following three elements represent the novelty of this proposed research framework:

- (i) Efficient memory management: this paper avoids any kind of bottleneck situation at the GPU end through the deployment of a memory-efficient variant of DenseNet-121 [19], unlike the preceding works.
- (ii) Bi-directional Sliding operations: learns bi-directional non-prehensile manipulation (left and right direction sliding) in contrast to unidirectional pushing in the existing approaches.
- (iii) Additional overhead avoidance: our framework avoids any kinds of additional overheads incurred by previous approaches due to the use of object segmentation and singulation techniques.

There are some existing mini production line setups, i.e., robotic arms and conveyor belts [20,21], available on the market for pick-and-place tasks but they are completely hard-coded and do not have any learning capability unless linked with a learning-based frame such as the one proposed in this paper.

With the approach described in the above-mentioned manner, the problem can be seen as a labeling problem where every pixel is labeled. Every pixel in the current state correlates with the prehensile and non-prehensile robotic manipulations. These manipulations are carried out at the three-dimensional location of the pixel in the current state. In the case of prehensile motion, the pixel location will be considered as the center point of grasping, whereas in the case of non-prehensile robotic manipulation, it will be taken as the initiation point of the left or right-slide. We train a fully convolutional network and supply it with the RGB-D images of the current scene of the workspace and obtain a pixel-wise prediction table as the output. This pixel-wise prediction table helps the agent select the appropriate pixel for robotic manipulation. These pixel-wise predictions procedures, also known as fully convolutional action-value functions [12], provide us with the expected future reward values for all the pixels whereby the pixel predicted to have the highest expected reward is chosen for robotic manipulation on the three-dimensional surface. These pixel-wise action-value functions make our agent learn and perform three different robotic manipulation policies after just a few hours of training.

In summary, our overall contribution is a data-driven framework that involves interlinked prehensile and non-prehensile robotic manipulations learned and performed through end-to-end deep networks based on the concept of pixel-wise parameterization. The main advantage of this work over others is that it deploys prehensile and non-prehensile manipulations in a sequential manner such that it outputs a high success rate for pick-and-place of regular and irregular objects in cluttered environments. Clutter can be seen as a randomly closely packed group of regular- and irregular-shaped objects. Cluttered objects are a common scenario in bin picking jobs in the industry. Similar to simple or sparse pick-and-place, bin picking where objects are to be picked from clutter has applications for implementation in all types of sectors such pharmacy, automotive, logistics and many others [22]. A few examples are: the world’s largest shock absorber manufacturer in Indiana, US requiring the picking of steel metal cylinders from clutter and their placement for further processing [23]; a leading door manufacturing company in the US requiring the packing of two different plastic parts after picking them from randomly organized clutter in two separate bins [23]; and the Italian sports gun manufacturer Benelli requiring small parts in clutter to be picked from bins and kitted in plastic boxes every day at its factory [23]. Responding to this worldwide demand, several research approaches have been presented for pick-and-place from clutter. The popular competition the Amazon Picking Challenge, which took place from 2015 to 2017 [24], was also focused on bin picking owing to its importance in industrial operations. Pixel-wise parameterization achieved through end-to-end deep networks is the key concept in our approach and will be explained in later sections. We present the results of various experimentations performed in a simulated environment designed using a V-REP simulator.

## 2. Related Work

Grasp planning’s history can be traced back to the beginning of the robotic manipulation era. Some studies consider the classification of grasps on the basis of their potential to restrict or limit the mobility of the object [25], while some other studies involve the modeling of contact forces and their opposing relation to grasping [4,26]. Some approaches work with pre-computed grasp poses at runtime by linking them with the point cloud data being used for object pose estimation [27]. These pre-generated grasps for translating the abovementioned approaches to real-life environments are usually created from databases of 3D object models [28]. A such, they require detailed knowledge beforehand, such as the poses, shapes, contact points, etc., of the objects under consideration, which is almost impossible to guarantee when dealing with novel objects in real-life environments. Aside from model-driven studies, various data-driven approaches have also recently emerged. Several studies have been carried out to train model-independent grasping policies in which an agent learns to detect potential grasps without having any prior object knowledge of factors such as the object’s pose, shape, contact points, etc. These policies are trained by learning the visual features instead [5,12,13,15,16,29]. Studies such as [5] present the idea of using pre-trained models on other robotic manipulations to decrease the learning effort while learning the target robotic manipulation (for instance, a model pre-trained on poking trying to learn grasping). In [12], deep networks were used to predict pixel-based affordances in order to make agents learn grasping policies. Similar to these studies, the approach presented in this paper is model-independent but involves the learning of a sequence of prehensile and non-prehensile robotic manipulations, leading to an efficient pick-and-place agent.

Non-prehensile motion can be dated back to the 1970s [30]. Most approaches presented in the early days tackled the task by modeling it based on frictional forces [7,8]. While these approaches motivated the research in this area, their assumptions while modeling the task, such as variability and the non-uniform distribution of friction, were not good enough to hold in real-world settings [10]. This caused these kinds of non-prehensile robotic techniques to fail in a real-world setting by making inaccurate predictions. Data-driven approaches to learn and perform non-prehensile robotic manipulation have also been presented [31]. Most of these data-driven approaches focused on training the agents to consider one object at a time. It is still a complex challenge to model clutter-based non-prehensile manipulation and develop solutions that can be proven effective in real-world scenarios.

Significant benefits can be obtained by combining both prehensile and non-prehensile approaches. The study presented in [2] is a pioneering model-driven approach to performing a push–grasp operation, a robotic manipulation in which grasping is performed within non-prehensile manipulations. With this method, grasping chances are increased. The study targets clutter-based workspace and it also focuses on achieving the ability to move objects in clutter. However, this whole technique requires domain knowledge beforehand, i.e., it is completely handcrafted. In contrast, the data-driven approach in this paper requires no domain knowledge while learning through self-supervision. There are some other approaches where grasping policies are already known and the task is to move objects using non-prehensile robotic manipulation to certain locations. These certain locations are locations where grasping can be performed according to the grasping policies known beforehand [9,32]. A reinforcement learning-based approach has been presented in [18] where the agent is trained to learn policies based on handcrafted features. The agent must choose between prehensile and non-prehensile manipulation in order to perform a pick-and-place task in a cluttered environment. The flow of their approach can be seen as first performing segmentation on RGB-D images received from the sensor. Through the segmentation, objects are identified. Once objects have been identified, multiple instances of actions are sampled (prehensile and non-prehensile manipulation) for each object. For each sampled instance, hand-crafted features are extracted and, finally, an action instance is performed that is predicted to have the maximum expected reward. The limitations to this approach are that it is trained for mostly convex objects only. The non-prehensile policy or pushing policy involved requires handcrafted features such as prediction of the motion of the pushed object. This prediction is made with the help of a simulator. The future effect of this pushing policy on the prehensile manipulation also needs to be known. In [33], the authors present two linear push policies to push objects away from the walls in bin-picking. Singulation techniques through reinforcement learning have been also presented but are limited to regular-shaped objects and unidirectional pushes [34,35]. Some approaches in the recent past have also been presented where pixel-wise-parameterization techniques have been used for the robotic manipulation of only regular-shaped objects, but mostly, they did not address non-prehensile motion [36] or movement in a limited and unidirectional manner [12,37,38]. In such approaches, memory management is a key limitation to be addressed. In contrast, the approach presented in this paper involves policies that are trained with end-to-end memory-efficient deep networks through pixel-wise-parameterization for prehensile and bidirectional non-prehensile robotic manipulation. These policies do not require or assume any kind of object knowledge of factors such as pose, shape, contact points, etc. Our results show that our approach performs successfully in various test cases involving many regular and irregular objects in clutter.

## 3. Methodology

**Figure 2:**
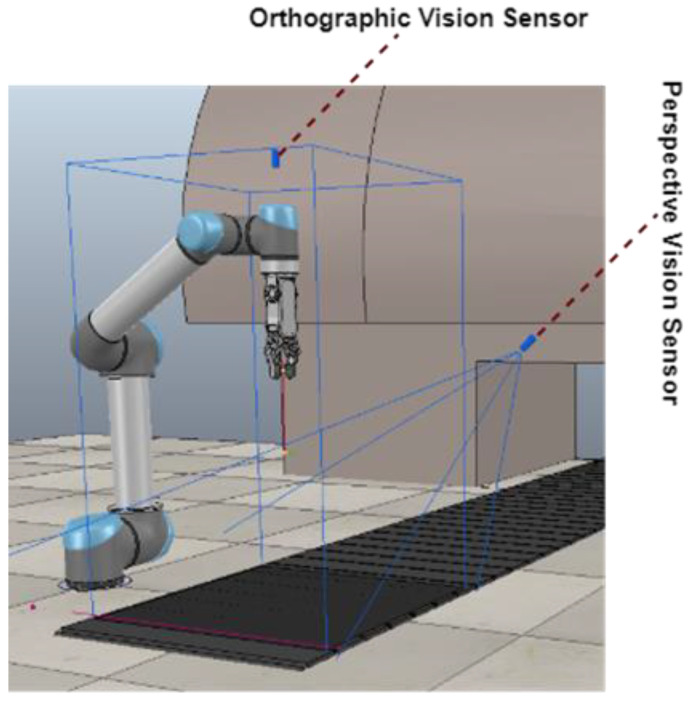
Location and field views of vision sensors in the simulated setup.

**Figure 3:**
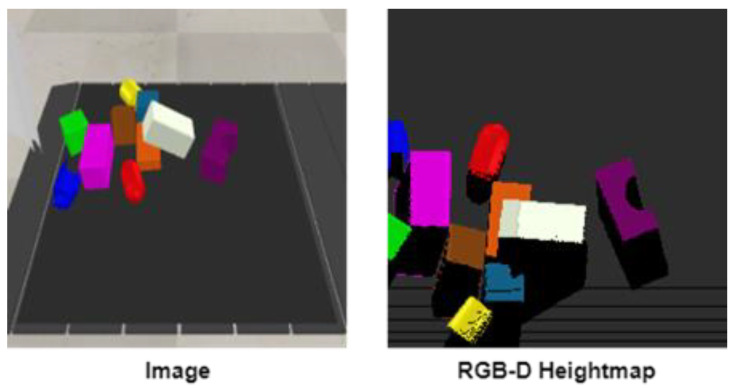
A sample of current view image from the workspace at timestep *t* and its generated RGB-D 224 × 224 pixel heightmap representation of the state *s_t_*.

**Figure 4:**
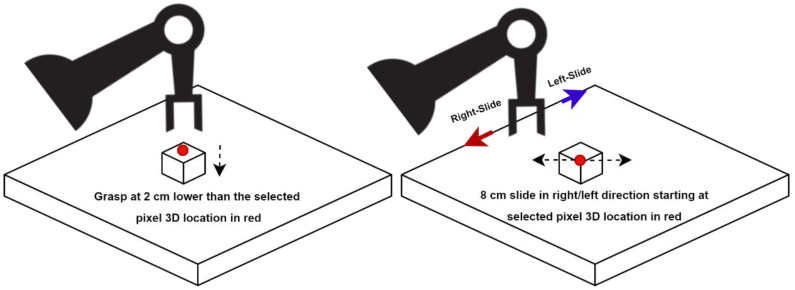
Grasp action (**left**) and left-slide and right-slide actions (**right**).

**Figure 5:**
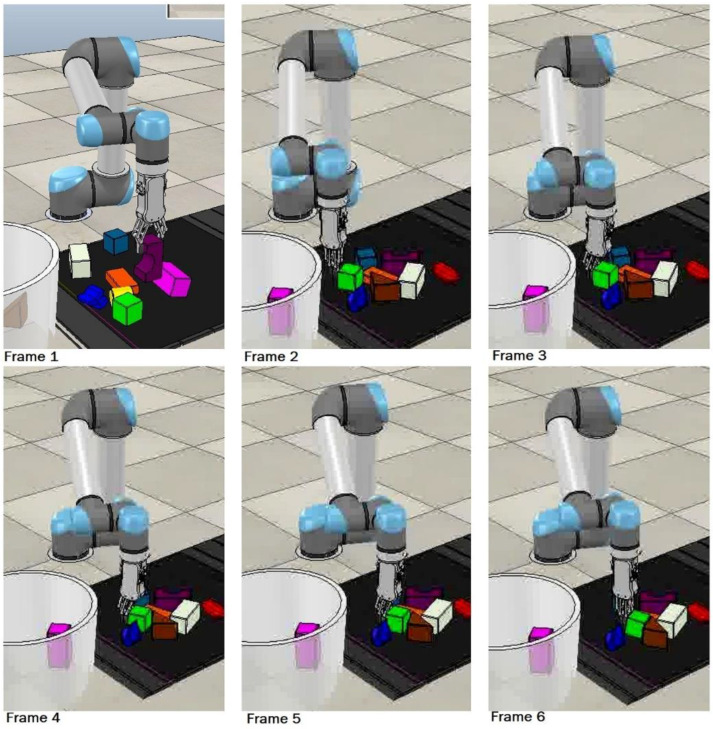
Frame 1 shows the grasping action whereas Frame 2 to Frame 6 show the sliding action being performed on the green square object.

In our framework, the agent learned to perform picking of regular and irregular-shaped objects in clutter from a conveyor belt and to place them in a bin with the help of a series of prehensile and non-prehensile robotic manipulation operations. In reinforcement learning, an agent is the entity that uses a policy to maximize the expected return gained from transitioning between states of the environment. In our case, the robotic arm UR5 can be seen later as an agent interacting with the environment, which is the workspace. We used reinforcement learning where a policy is learnt to solve the task through sequential decision-making. As this is a learning-based data-driven framework, the success lies in learning to predict the most suitable pixel of the workspace for the grasping and sliding actions. The secondary success, as shown by the results, is the successful learning of synchronization between the prehensile (grasping) and non-prehensile (sliding) actions. The RL agent learns to perform such non-prehensile manipulations which lead to successfully rewarding prehensile manipulations in the near future and vice versa, whereas the grasping pose is considered static in our work, always from the top angle, and robotic arm motion planning is dynamic and controlled by the Open Motion Planning Library (OMPL) built into the V-REP simulator. The overall success of the framework was calculated by measuring the attempts taken to clear the workspace of all the objects, which could only be achieved efficiently if the agent was able to learn the abovementioned factors and the policy converged successfully. According to the design of the problem, an MDP was formulated. The basic elements of the MDP are as follows:

- s ∈ S (RGB-D heightmap), where RGB-D heightmap represents the heightmap generated from the current scene of the workspace at any time instance. In this approach, we used vision sensors to obtain the view of the current state of the workspace. Two types of vision sensors were used in the environment: an orthographic projection vision sensor and a perspective projection vision sensor. The basic difference between these two is their field of view. Orthographic projection vision sensors have a rectangular projection view, whereas perspective projection vision sensors have a trapezoidal field of view. Figure 2 shows the location and fields of view of these vision sensors in our designed simulation setup. We captured RGB-D images through these fixed position vision sensors and aggregation was performed to gather maximum RGB and depth information. Basically, an RGB-D image constitutes an RGB image and its corresponding depth image. A depth image contains information relating to the distance of the surfaces of scene objects from a viewpoint, which, in our case, was the height-from-bottom information. While an RGB image has three channels (red, green, and blue), the depth image has one channel in which each pixel is in relation to the distance between the image plane and the corresponding object in the RGB image [39]. From these RGB-D images, we created RGB-D heightmaps. We followed the method of heightmap computation from the RGB-D images used in [12] where the RGB-D data are projected onto a 3D point cloud, which is then orthographically back-projected upwards in the gravity direction, leading to a heightmap representation of the workspace with both color (RGB) and height-from-bottom (D) channels. Thus, each RGB-D heightmap representation was taken as the state *s_t_* at time *t*, as shown in Figure 3. The RGB-D heightmaps created through this process comprised 224 × 224 pixels—the size of our workspace. Each pixel in the heightmap represents a unique three-dimensional location in the physical workspace.

- a ∈ A: (grasp, left-slide, and right-slide), where grasp, left-slide, and right-slide are three unique available actions in this MDP. We designed each robotic manipulation as a distinct action, i.e., grasping (prehensile), left-slide and right-slide (non-prehensile). The motive behind choosing actions from both categories is that non-prehensile manipulations will assist and lead to prehensile manipulation. Each action $a_{t}$, at a given time *t*, will be performed at a three-dimensional location $p^{\prime }$ corresponding to a pixel p of the RGB-D heightmap or state $s_{t}$. This formulation can be seen as $p\to p^{\prime }\;\in s_{t}$, where $p^{\prime }$ is the mid-point of the grasping. As we have top-mounted vision sensors, our grasping will be carried out from above using an RG2 gripper [40], which is a parallel-jaw grasper that went approximately 2 cm deeper than the $p^{\prime }$’s z coordinate, as shown in Figure 4, for a firm grasp. On the other hand, if left-slide or right-slide is to be performed, $p^{\prime }$ is the three-dimensional location that will serve as the starting point of an approximately 8 cm linear straight push with the tip of the closed parallel-jaw grasper in the left or right direction of the gripper, respectively, as shown in Figure 4. The left-slide and right-slide actions will be performed with reference to the robotic arm and the red and blue arrows show the direction of the right-slide and left-slide, respectively, in Figure 4. For instance, if we imagine ourselves as the robotic arm, our right hand points in the direction of the right-slide and our left hand points in the direction of the left-slide. These grasping and sliding actions can be seen in Figure 5, where Frame 1 shows the grasping action and, upon close inspection, Frames 2 to 6 show the sliding action where the green cuboid object is being slid/pushed by the robotic gripper. To accurately assess the sliding action, one can notice the distance between the green cuboid object and the brown triangular object reducing frame by frame, especially from Frames 4 to 6.

- r ∈ R (1, 0.8, 0.5, 0), where 1, 0.8, 0.5, and 0 are the unique rewards in our rewarding scheme. A reward scheme in a reinforcement learning approach is designed based on possible outcomes. In our approach, we considered four possible outcomes. Firstly, the object is grasped and placed at the designated location successfully; secondly, the object is grasped successfully but falls during displacement to the designated location (in the basket); thirdly, a successful left- or right-slide is performed; and finally, any other outcome occurs. For the first outcome, the agent is awarded 1; for the second outcome, which is verified by an infrared sensor between the gripper jaws, the agent is awarded 0.8; and for the third outcome, verified by finding changes in the workspace by comparing current and previous heightmaps, the agent is awarded 0.5. Any other outcome is considered a complete failure, so the agent is awarded 0 as a reward.

According to the flow of our designed MDP, our agent, i.e., the robotic arm, at any given state *s_t_* at time *t*, according to the policy *π*, can choose and execute an action a_t_ from the list of available actions, ending up in a new state *s_t_*_+1_. It then receives a reward *r_at_(s_t_, s_t_*_+1_), meaning a reward is earned after performing action *a* at time *t* during the transition from state *s_t_* to state *s_t_*_+1_. The goal of this task is to discover the optimal policy π* through which we can achieve the goal of maximization of the expected rewards in future, given by Equation (1) as follows:

(1) $$R_{t}=\Sigma_{i=t}^{\infty }\;\gamma R_{ai}\left(s_{i},s_{i+1}\right)$$

where γ denotes the discount factor, usually ranging between 0 and 1, thus making the agent learn at a faster pace. In this paper, we designed our approach based on an off-policy model-free temporal difference learning algorithm known as Q-learning. We aimed to achieve convergence of a policy *π*, which is greedy in nature and performs action selection based on the maximization of the action–value function, also known as the Q-function. A Q-function, which can be described as *Q_π_*(*s_t_*, *a_t_*), is actually our tool that predicts the expected reward for performing an action *a_t_* while being at state *s_t_* at any time *t*. We can formally see our Q-function working in Equation (2).

(2) $$Q\left(s_{t},a_{t}\right)\leftarrow Q\left(s_{t},a_{t}\right)+\alpha \left[R_{at}\left(s_{t},s_{t+1}\right)+\gamma Q\left(s_{t+1},argmax\left(Q\left(s_{t+1},a_{t}^{\prime }\right)\right)-Q\left(s_{t},a_{t}\right)\right)\right]$$

where α denotes the learning rate and $a^{\prime }$ denotes the list of all available actions.

### 3.1. Q-Function Design

**Figure 6:**
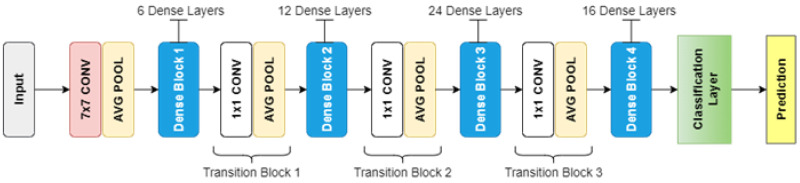
DenseNet-121 general architecture.

We used deep Q-networks for Q-function approximation to obtain the state-action values that predict the expected future rewards for carrying out an action $a_{t}$ at a state $s_{t}$ [41,42]. The Q-function that we designed is a combination of feed-forward fully convolutional networks (FCNs) [43]. For more efficient learning of features, we created a distinct FCN for each of the available actions, which are denoted as {$FCN_{Grasp},\;\;FCN_{LeftSlide},\;\;FCN_{RightSlide}$}. Each of these FCNs is supplied with state *s*, which is represented as a heightmap, processes it, and generates a pixel-wise map as the output. The values in this map are the Q-values. As mentioned earlier, the state is represented by a heightmap of 224 × 224 pixels in accordance with the workspace size and the dense pixel-wise map generated by the FCN as the output is also of the same 224 × 224 size. Each value in this dense pixel-wise map corresponds to each corresponding pixel in the heightmap representation of the state. Each value in this pixel-wise map is a prediction of the expected future reward the agent will earn after performing the corresponding action at $p^{\prime }$, with the three-dimensional location corresponding to the pixel p. All three FCNs {$FCN_{grasp},\;FCN_{LeftSlide},\;\;FCN_{RightSlide}$} have the same architecture, i.e., DenseNet-121 [44], whose general architecture is presented in Figure 6.

**Figure 7:**
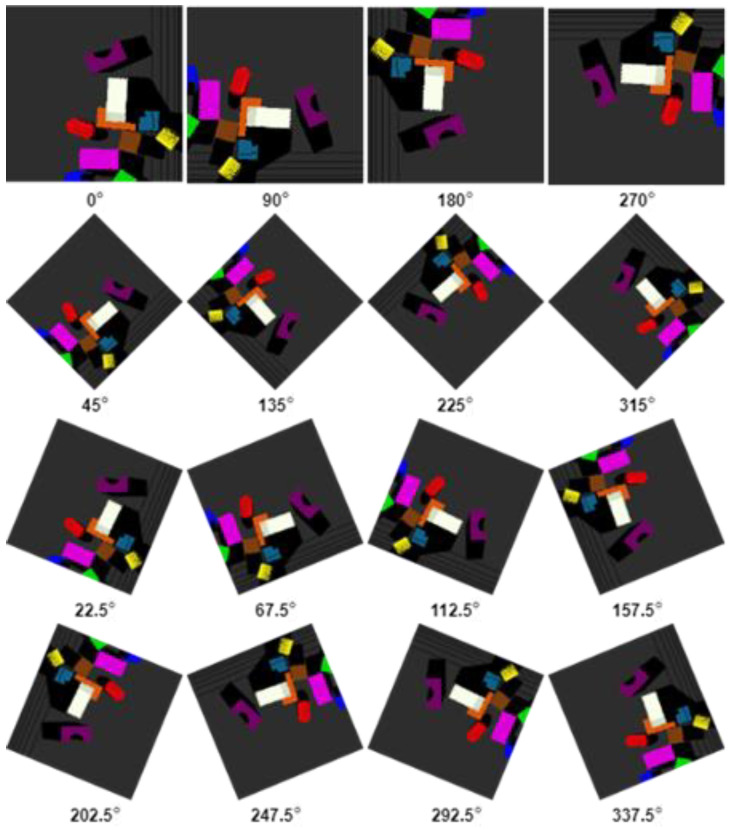
RGB-D heightmap rotations at 90 degrees (first row), 45 degrees (first and second row), and 22.5 degrees (all four rows).

The main difference between other traditional convolutional networks and DenseNet is the number of connections. A traditional convolutional network comprising *L* layers has *L* connections, which means every layer is connected to its immediate successor layer; on the other hand, DenseNet comprises $L\left(L+1\right)/2$ connections, where any layer will obtain the feature maps from all its predecessor layers as inputs and will supply its own feature map to all its successor layers as their input. This is mainly the reason behind adopting the DenseNet architecture in our approach. With the help of DenseNet, feature reuse is enabled, feature propagation is made better, the vanishing gradient problem is addressed, and parameter reduction is achieved. However, at the same time, all these benefits lead to quadratic growth of the features as the network becomes deeper and deeper, which results in choking of whole network and the formation of a bottleneck scenario. Therefore, we addressed this issue in a different manner, which will be stated in future sections. DenseNet-121 was used in two manners: trained from scratch and also pre-trained on ImageNet [45]. The structure of DenseNet-121 was extended with two additional 1 × 1 convolutional layers along with rectified linear unit (ReLU) activation function [46] and batch normalization [47] procedures. We used the multimodal approach shown in Figure 7 as in [12], and within an FCN, we created two trunks of DenseNet-121; one was supplied with color channels (RGB) from the heightmap representation of the state, and the other trunk was supplied with clones across the three-channel depth (DDD) of the heightmap, which was normalized by subtracting the mean and dividing by the standard deviation. This cloning of depth across channels was performed to exploit the weights pre-trained on ImageNet RGB images. Features learned through both these trunks were concatenated and supplied to the extended 1 × 1 convolutional layers.

To enhance the learning of features for robotic manipulations, we brought in the rotation of heightmaps. Initially, we rotated the input heightmaps by 90 degrees, leading to 4 rotated heightmaps in total. Later, we experimented with rotating heightmaps at 45 and 22.5 degrees, leading to 8 and 16 rotated heightmaps in total, respectively. Figure 7 shows all three rotation modes; the first row shows the 90-degree rotations, adding the second row shows the 45-degree rotation, and all the rows together show the 22.5-degree rotations. The results show that features were learn better when heightmaps are rotated at 22.5 degrees, generating 16 heightmaps, so that the factor of different orientations is handled well. In this way, each FCN is supplied with 16 rotations of heightmaps and, in turn, outputs 16 dense pixel-wise Q-values maps. So, the total output generated from all the three FCNs {$FCN_{Grasp},\;\;FCN_{LeftSlide},\;FCN_{RightSlide}$} is 48 dense pixel-wise maps, out of which 16 are for grasping, 16 are for the left-slide, and 16 are for right-slide. Simply put, the 48 maps are an extension of the 16-rotation scheme instead of being two different schemes. Processing was carried out for all three actions (grasp, left-slide, and right-slide) at parallel; each action’s processing obtained 16 rotated pictures, and collectively, they amounted to 48 (16 + 16 + 16). In other words, when we consider a single action, it is 16 inputs and 16 outputs, but when we collectively consider all three action, it becomes 48 inputs and 48 outputs. In all these Q-values from the total 48 dense pixel-wise maps, the maximum one is selected for the particular action corresponding to the FCN it is generated from. Action was performed on $p^{\prime }$, the three-dimensional location of the pixel p whose corresponding maximum Q-value was selected. This is depicted as in Equation (3).

(3) $$max\;Q\left(s_{t},a_{t}\right)=max\left(FCN_{Grasp}\left(s_{t}\right),FCN_{LeftSlide}\left(s_{t}\right),FCN_{RightSlide}\left(s_{t}\right)\right)$$

In our pixel-wise affordances approach, these FCNs were used as Q-function approximators. FCNs have proven their worth in pixel-wise scenarios through efficient computation mechanisms. In each forward pass in our network, 2,408,448 Q-values are computed in a time-efficient manner. This total number of Q-values is calculated as each pixel-wise map is of 224 × 224 size, and such 48 pixel-wise maps are generated in every forward pass (224 × 224 × 48). The convergence of our policies was achieved with a lower training data requirement due to this pixel-wise parameterization and the involvement of rotation functionality (helping with learning various orientations), thus enabling the agent to learn at the maximum rate.

### 3.2. Training

**Figure 8:**
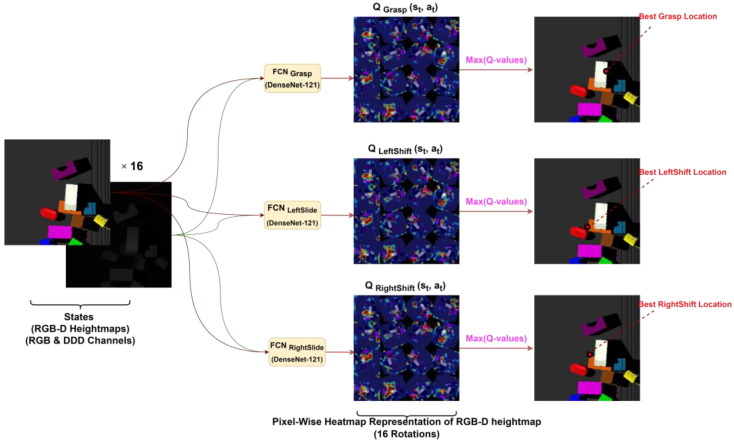
The flow of the whole approach is shown in this figure. RGB-D heightmaps comprising RGB and DDD channels generated as state representations are rotated by 22.5 degrees and all 16 rotations are fed to all three FCNs {*FCN_Grasp_, FCN_LeftSlide_, FCN_RightSlide_*} based on DenseNet-121 architecture. A pixel-wise map of affordances is generated by each FCN against each rotation of RGB-D heightmap. This map constitutes Q-values, shown in the heatmap format. The maximum Q-value is chosen out of all 48 pixel-wise maps for relevant action to be performed.

**Figure 9:**
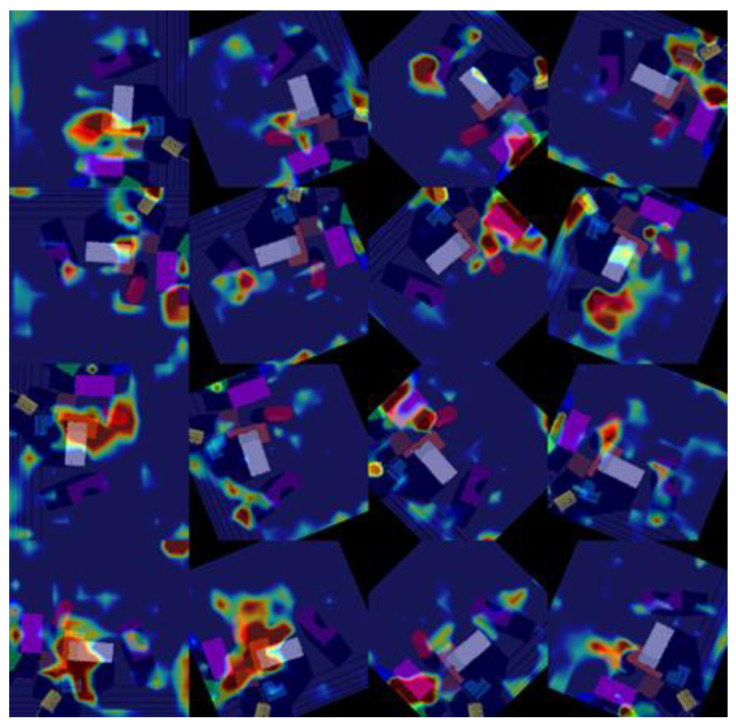
Heatmap visualizations of pixel-wise maps of Q-values generated against 16 rotations of an RGB-D heightmap at 22.5 degrees.

In each iteration, after receiving RGB-D images from the vision sensors, the heightmap representations are generated as the current state. The rotation function, shown in Figure 7, is then performed on the heightmaps, the rotated heightmaps are fed to the network, and pixel-wise maps are generated as outputs in return, as shown in Figure 8. As the same workspace state is being supplied, all three FCNs’ outputs are shown on the same workspace image, but the difference or the actual output is the RED dot in all three output pictures labeled as the best location for each action. For instance, the top image shows the best location (red dot on the left edge of the white rectangular object) for the grasp action according to the 16 pixel-wise Q-value maps generated by its *FCN_Grasp_*. The middle image shows the best location (red dot on almost the lower corner of the orange rectangular object) for the left-slide action according to the 16 pixel-wise Q-values maps generated by its *FCN_LeftSlide_*. The bottom image shows the best location (red dot on the upper corner of the orange rectangular object) for the right-slide action according to the 16 pixel-wise Q-value maps generated by its *FCN_RightSlide_*. The highest Q-value of these all three pixels (locations) will be selected for the corresponding action. These pixel-wise maps can be visualized in heatmap format as shown in Figure 9, where hot regions depict higher Q-values. For the backpropagation, we used the Huber loss function [48], which can be considered as a trade-off between the mean square error (MSE) and mean absolute error (MAE). In our backpropagation scheme, gradient passing was only performed through a particular FCN and pixel p, which gave the maximum Q-value for action selection, whereas 0 loss was backpropagated for all the remaining pixels. FCNs were trained using stochastic gradient descent with momentum [49], which is considered more efficient and rapid compared to traditional stochastic gradient descent (SGD). The traditional SGD calculates noisy derivatives of loss, whereas the SGD with a momentum variant involves exponentially weighted averages. The traditional SGD is outperformed by the momentum variant because these exponentially weighted averages are much closer to the actual loss compared to the SGD’s noisy values. Therefore, an SGD with momentum helps gradient vectors to move in the right direction, leading to faster convergence. While training our network, the values of momentum, learning rate, and weight decay were set at 0.9, 1 × 10^−2^, and 1 × 10^−3^, respectively. In our training phase, we also used experience replay, where the agent keeps a record and reuses its experiences from the past. We adopted the stochastic prioritization approach developed in [50] for experience replay. To address the exploration vs. exploitation dilemma, we deployed epsilon-greedy action selection, where the epsilon value was 0.3 at initiation and gradually decreased down to 0.1. Our framework was developed and trained on a NVIDIA GeForce RTX 2080/PCIe/SSE2 with dedicated 8 GB memory using an Intel^®^ Core™ i7-9700K CPU @ 3.60 GHz × 8 processor along with 16 GB RAM. The development and training of the models was performed in PyTorch version 1.7.1 using CUDA version 11.4, cuDNN version 8.2.2, and NVIDIA Driver version 470.103.01.

DenseNet is one of the most efficient architectures due to its maximized links leading to maximum feature reuse. However, at the same time, if it is not managed properly, due to its contagious convolutional operations its features, maps can start growing quadratically, thus creating a bottleneck in the GPU. Therefore, we implemented a memory-efficient variant as in [19], where shared memory schemes are used for concatenation, batch normalization, and gradients.

**Figure 10:**
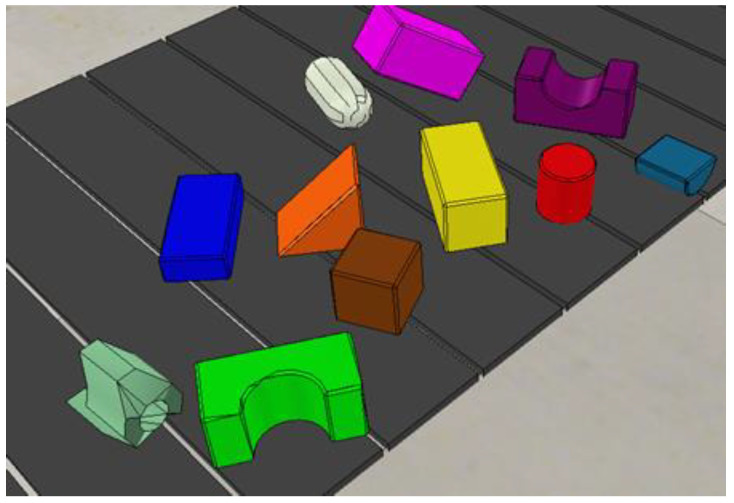
Ten regular and irregular 3D objects randomly used in the approach.

In our simulated environment, all agents are trained via self-supervision through trial and error. At the start of the training process, the workspace on the conveyer belt has N number of regular and irregular objects in a random clutter. The agent starts trying to pick and place these objects and keeps on doing so until the workspace is cleared of all the objects. Once all objects have been placed inside the basket, one iteration is completed and a new random clutter of objects appears in the workspace for the next iteration, and the process goes on. In our training, we kept the value of N = 10 for training, which means a random clutter of 10 different regular and irregular 3D objects (shown in Figure 10) in each iteration.

### 3.3. Simulation and Motion Planning Specifications

The Virtual Robot Experimentation Platform (V-REP) is a 3D robotic simulator with integrated development and coding support [51]. It also has the physics engines Bullet and ODE, providing a real-time experience of the objects involved in the simulation [52,53]. The API and threaded/non-threaded Lua scripting functionalities make it a reasonable option for combining multiple platforms such as python, java, C++, etc., for experimentation. Using this API, we were able to make our python-based RL agent communicate with the Lua-scripted simulated environment.

**Figure 11:**
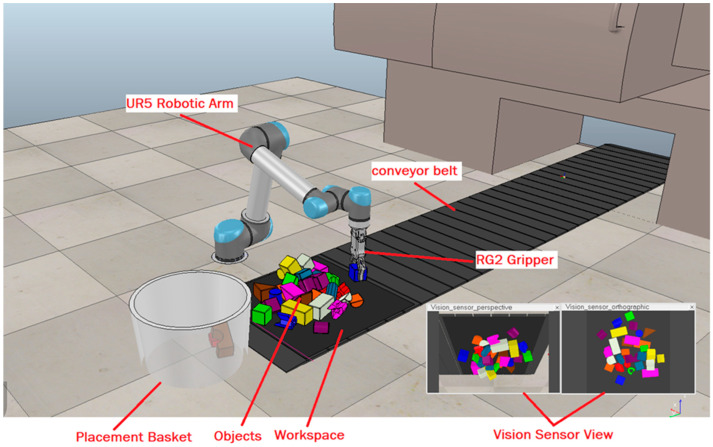
Whole simulation scene designed in V-REP.

V-REP provides various motion planning calculation modules including the forward and inverse kinematics module. Forward kinematics means using kinematic equations, taking joints parameters as inputs, to calculate the position of an end-effector, whereas inverse kinematics is the reverse process, calculating joint parameters for the given position of an end-effector [54]. The collision detection module is another important module in V-REP used to highlight collisions if there are any. We used the UR5 robotic arm [55], a well-known cobot, for our approach, which is a 6-degrees-of-freedom robotic arm, and we used an RG2 gripper for grasping and sliding purposes. The whole simulation scene designed in V-REP can be seen in Figure 11. A conveyor belt was used in our experimentation. A ray-type infrared sensor is installed at the workspace end of the conveyor belt. The conveyor belt keeps moving, and as soon as the clutter of objects reaches the infrared sensor and breaks the ray, they are detected by the sensor. The sensor resultantly sends a signal to the conveyor belt controller to stop and to the vision sensor controller to capture the workspace RGB-D images for processing. As the workspace clears, the infrared sensor ray is reinstated and the conveyor belt starts moving again, thus bringing the next clutter, and in this way, the cycle continues.

We explored a number of options for performing our motion planning task such as OpenRave and Trajpot [56,57]. However, the best fit with our simulated environment was the Open Motion Planning Library (OMPL) [58] as it provided us with a certain degree of customization and control over the motion planning algorithms. The OMPL contains a number of geometric and control-based planners. Some of the many sampling-based planners available are Single-query Bi-direction Lazzy (SBL), Expansive Space Trees (EST), Rapidly exploring Random Trees (RRT), and the Probabilistic Roadmap Method (PRM), along with their many variants. In a similar way, many commonly used path planning state-spaces are readily available in an implemented form. The planner we used for motion planning and path calculations is a single-query planner, a bidirectional variant of Rapidly exploring Random Trees (RRT), known as RRT-Connect [59]. The core logic behind RRT-Connect is to develop two RRTs, one at the start point and the other at the end, and then, connect them. Due to this, the RRT-Connect planner tends to outperform the RRT planner.

## 4. Experimental Results and Discussion

In this section, we present a series of experiments carried out in order to see to what extent our goals can be achieved. Through these experiments, we assess the learning ability of the agent to perform pick-and-place tasks, the efficiency of prehensile manipulation, and the role of non-prehensile manipulation in doing so.

**Figure 12:**
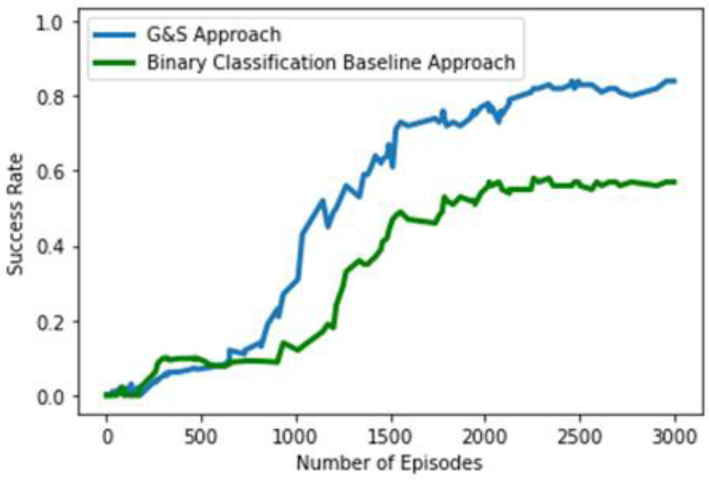
Performance comparison of original G&S approach and binary classification baseline approach.

To evaluate the performance of our agent, we compared it with a deep learning baseline framework. We name our original approach G&S, which is the abbreviation for grasp and slide. The baseline approach is quite like our original approach. It is also designed around the idea of pixel-wise parameterization, but it is a deep learning-only-based supervised binary classification approach, i.e., there is no RL component. The same method of state representation and the same number of actions are formulated. As the number of actions is the same in both cases, the same number of FCNs are used. Like the concept of the original approach, each FCN will generate a dense pixel-wise map of affordances for the corresponding action. The only difference will be that the FCNs will be trained with binary classification (0 and 1) in a supervised manner and will generate a dense pixel-wise map of affordance values between 0 and 1. The labels for supervision will be generated on the basis of the success of grasping and sliding so that if grasping is successful, leading to placement of the object in the basket, it will be labeled as 1, and otherwise, as 0; moreover, if a change is detected in the workspace scene after left- or right-slide action is performed, it will be considered successful and labeled 1, and otherwise, as 0. Backpropagation was also performed in the same manner as in our G&S approach, as gradient passing was only performed through a particular FCN and pixel p, which gave the maximum corresponding value in the pixel-wise dense map for action selection, whereas 0 loss was backpropagated for all the remaining pixels. This supervised learning approach policy is considered greedy as it will go for the action with the maximum pixel-wise affordance value at any timestep. This baseline approach revolves around the concept of a pixel-wise affordance-based grasping approach designed in [12], but for an accurate comparison, we used similar DenseNet-121 [44] architecture and weights from the pretraining on ImageNet [45]. The comparison of the performance of our agents trained from scratch using the G&S approach and the baseline binary classification approach can be seen in Figure 12. In this comparison, both agents were trained for 3000 episodes, where each episode involved 10 randomly chosen regular- and irregular-shaped objects and ended when all the objects were placed, or at least ten actions consecutively failed to bring any change to the workspace. The blue line represents the success rate of our G&S approach, which is around 84%, and next to it, the green line represents the success rate of the binary classification approach, which is around 57%. This measure of success rate is estimated as the number of successfully placed objects in the episode over the total number of actions performed in the episode. A probable explanation for the reduced efficiency of our baseline binary classification approach may be its greedy approach. Instead of building and following strategies based on future perspectives as our G&S approach does through reward schemes, it focuses only on selecting actions based on the maximum pixel-wise affordance value at the current timestep.

**Figure 13:**
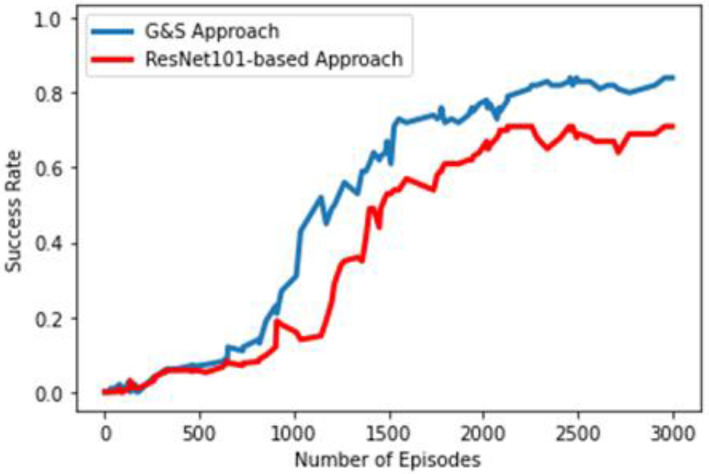
Performance comparison of original G&S approach and ResNet-based approach.

We also designed another variant of our G&S approach, and the only difference between the original and this variant is that it uses the ResNet-101 architecture pre-trained on ImageNet [45] instead of DenseNet-121. ResNet [60], also known as a residual network, is made up of residual blocks. It was designed to tackle the fact that every network has a certain threshold value for its depth; as more and more layers pile up over the top of the network, its performance starts falling due to the notorious vanishing gradient problem. In the vanishing gradient problem, during backpropagation, as the gradient is transmitted through the depth, i.e., previous layers, it shrinks to an infinite level due to repetitive multiplications. ResNet resolves the vanishing gradient problem by introducing identity shortcut connections, also known as skip connections, which skip one or more layers, thus providing better gradient flow. The performance comparison of our original G&S approach based on DenseNet-121 pre-trained on ImageNet and its variant based on ResNet-101 pre-trained on ImageNet is shown in Figure 13. The original approach outperforms the ResNet-based variant by a margin of around 13%. The possible reason may be that ResNet performs a summation of feature maps of the layers, but DenseNet performs concatenation of feature maps, thus allowing for feature reusability at the maximum level. Whereas the identity shortcut connections restrict the representation capacity in ResNet, the multi-layer concatenation of feature maps through $L\left(L+1\right)/2$ links among the *L* layers in DenseNet enables it.

**Figure 14:**
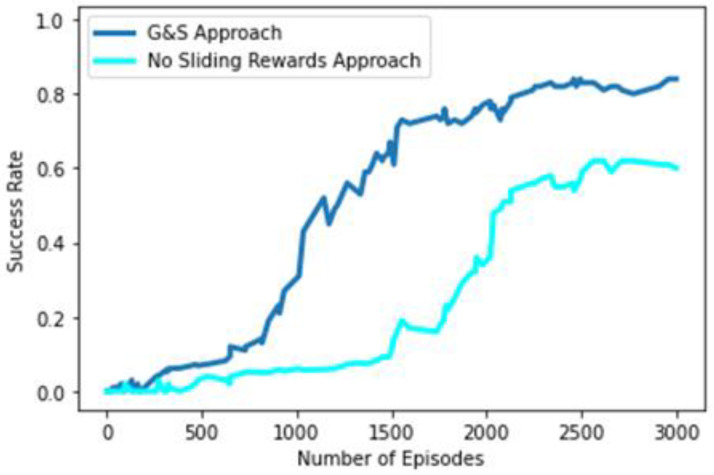
Performance comparison of original G&S approach and no-sliding-rewards approach.

As the motive of our study is to train our agents in such a manner that they can learn the pick-and-place task through a combination of prehensile (grasping) and non-prehensile (left-slide and right-slide) robotic manipulations, we explored the role of non-prehensile actions in the convergence of the policy being learnt through trial and error under self-supervision. To understand the contribution of sliding manipulations to the overall learning of the pick-and-place task, we trained another variant of our original G&S approach with the difference that reward allocation for non-prehensile manipulations (left-slide and right-slide) was discontinued. Rewards were only awarded for prehensile manipulations (grasping) so that whenever an object was moved, the agent earned no reward. Figure 14 shows the performance of the original G&S approach in the blue line and the reward-less sliding variant in the cyan line. Removing rewards for sliding manipulations shows performance degradation of around 22% in terms of success rate. This highlights the importance of the contribution of non-prehensile manipulations in the learning of agents. The delayed rising curve of the success rate depicts the slower pace of the agent in learning the policy because the non-prehensile manipulation rewards are not there; thus, the agent is, by default, learning a policy that is more grasping-oriented. In this situation the importance of sliding manipulation can only be learnt indirectly through the rewards earned through grasping performed in the future after sliding manipulation; therefore, a lower rate of improvement is indicated by the delayed rising curve compared to the original G&S approach.

**Figure 15:**
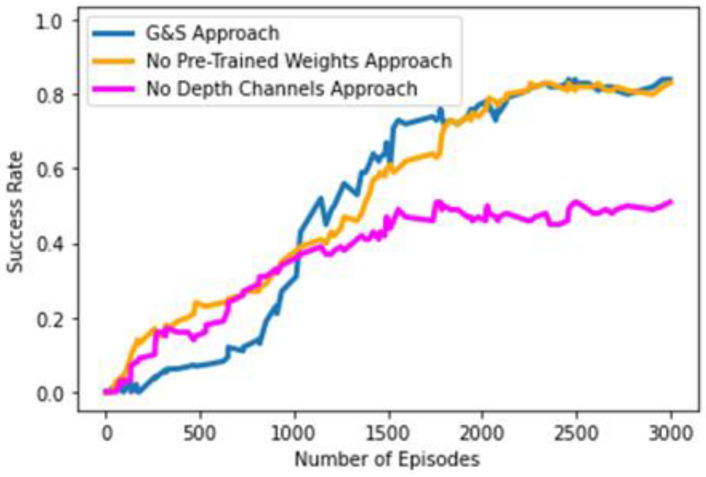
Performance comparison of original G&S approach, no-pre-trained-weights approach, and no-depth-channels approach.

In our G&S approach, we deployed DenseNet-121 architecture pre-trained on a popular ImageNet subset [61] comprising around 1,431,167 training, validation, and testing images in total belonging to around 1000 object classes. In our work, we also investigated the role and contribution of pre-trained weights, gained through training over ImageNet, in the learning process and the convergence of our learning policies by training a variant of our original G&S approach from scratch, in other words, involving no pre-trained weights. Figure 15 shows the success rates of both our original G&S approach in the blue line and its variant without pre-trained weights in an orange color. The results show that these pre-trained weights do not play much of a part in the process of learning the pick-and-place task, as the performance of both agents’ is similar. Usually, pre-trained weights are involved in an architecture to decrease training time and increase accuracy. However, this scheme of using pre-trained weights does not make much difference in our case. We surmise that the likely reason may be the different kind of pixels pattern in our RGB-D heightmaps generated by following the approach presented in [12] compared to those in the images of the ImageNet dataset. In Figure 15, one can see the success rate’s early curve increase in the orange line, representing the variant with no pre-trained weight. The delayed curve increase of the original G&S approach, represented by the blue line, can be seen in Figure 15. We suspect that the likely reason behind the delayed curve increase is the fact that the agent’s initial training steps were consumed to escape the local optima problem due to pre-trained weights.

Another element in our approach that is worth exploring is the depth information or the depth channels in our heightmaps. In Section 3.1, we explained that we cloned the depth across three channels to exploit weights pre-trained on ImageNet images. As such, as we already created a variant of our approach which is trained from scratch instead of using pre-trained weights, we removed the depth channels providing information regarding height from the bottom; then, for each FCN, we created only one trunk of DenseNet-121 to learn features from RGB channels instead of two trunks, because no second trunk was required to learn features from the cloned depth channels (DDD), and then, we further concatenated them with the features learnt from the RGB channels. In Figure 15, the magenta line represents the success rate of the variant of our G&S approach, which is trained from scratch without being provided with depth channels for feature extraction, and thus, no information regarding the height from the bottom. Excluding the feature learning from the depth channels led to performance deterioration as the success rate dropped to around 51% over the period of 3000 episodes. This clearly shows that the depth information or height-from-the-bottom information is quite crucial in the learning process of the agent and optimal convergence of the learning policies.

**Figure 16:**
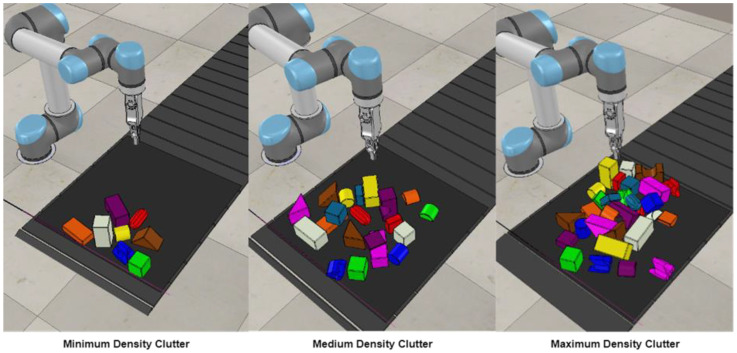
Three categories based on clutter densities.

Further, we also reviewed our G&S approach’s performance under unseen circumstances, which were not experienced during the training phases. As described in Section 3.2 during our training phases, the workspace on the conveyer belt has N number of regular and irregular objects in a random clutter and the agent starts picking and placing those objects and keeps on doing it until the workspace is cleared of all the objects. During the training phases, the number of random objects N was kept at 10. After training our agent for 3000 episodes consecutively on a clutter of 10 objects, we tested our agent by varying the clutter density. To bring variations in clutter density, we created three categories of clutter, as shown in Figure 16.

**Figure 17:**
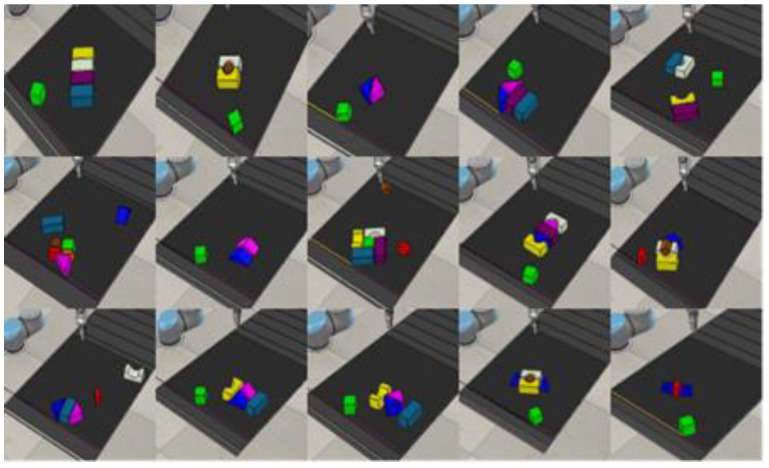
Complicated scenarios comprising 3 to 7 regular and irregular objects, designed for testing.

The first category is named the minimum density clutter, where regular and irregular objects are randomly distributed, and the number of objects ranges from 6 to 10. The second category is the medium density clutter, where the number of objects remains between 15 to 20 and they are distributed randomly. The third and last category, named the maximum density clutter, also comprises randomly distributed regular and irregular objects ranging from 25 to 30 in number. In addition to this, we designed several complicated cases comprising at least three and at maximum three objects, as shown in Figure 17, in order to test the behavior of our agent in these complicated scenarios. In these complicated scenarios, one or two objects were always kept free, whereas the remaining five to six regular and irregular objects were placed in some kind of locking manner so that these objects caused resistance while the agent tried to grasp or slide them.

The average performance results of these clutter variations and complicated test cases are shown in Table 1.

We can see in Table 1 that we compared the grasping success of our agent along with the overall success rate. This grasping success is calculated as the number of successful grasps over the total number of grasps performed in the whole episode. For instance, if 10 grasping actions were performed during the episode and 8 of them were successful, then grasping success will be calculated as 8/10 × 100 = 80%. The results show that our agent performs well in minimum and medium clutter density test cases, but in maximum clutter density, performance degrades a little in both overall success rate and grasping success. The major reason behind this degradation of performance is the factor of objects appearing in the workspace on the conveyer belt at random locations, and due to high density, these objects are placed on each other in random weak poses. This is also visible in maximum density clutter, as shown in Figure 16. As soon as the robotic gripper goes to grasp an object that is weakly posed on other objects, they all slip and fall, leading to failed grasping and degradation of the overall performance. In the case of complicated scenarios, we intentionally placed objects in locking poses such that it became difficult for the agent to pick and place them. For instance, in some places, there was not enough space for grasping or sliding, and at the same time, some objects were too wide to be grasped. These scenarios were met by the agent for the very first time, as the object arrangements were completely random and not hand designed. Another reason for the decrease in overall performance is the smaller number of objects in the scenario. For instance, if the test case has only three objects, the agent clears the workspace in four actions, which is a very good performance, but still, the overall success rate will fall to 3/4 × 100 = 75%. Therefore, we can say that the agent has truly shown its learning in our test cases in a very reasonable manner.

## 5. Conclusions and Future Work

In this study, we have presented a framework designed to enable intelligent and self-supervised industrial pick-and-place operations involving cobots. Our framework deals with the learning of both prehensile (grasping) and non-prehensile (sliding) robotic manipulations in a memory-efficient manner, therefore allowing the agent to learn and perform a sequential combination of prehensile and non-prehensile robotic manipulations to increase the efficiency and throughput of the pick-and-place task at hand. This approach deals with three unique actions and does not require any kind of prior domain knowledge or geometrical pose information like some previous approaches. In this our approach, we made our agent learn one prehensile manipulation—grasping—and two non-prehensile robotic manipulations—left-slide and right-slide. We used the pixel-wise parameterization method along with deep Q-learning to obtain pixel-wise maps of Q-values. The action with the highest Q-value is performed at the 3D location of the pixel that is predicted to have the maximum Q-value. Rewards are awarded accordingly for successful actions. Through the process of self-supervised trial and error, our agent learns and converges to the optimal policy, which enables the agent to perform successful non-prehensile manipulations at such moments where they lead to the possibility of successful prehensile manipulation in the future. Our agents learn these policies and perform successfully, as shown in our Section 4, where we show comparison results of different variants and baseline approaches and the test results for varying clutter densities and different test cases.

Thus, our approach shows many promising results, but at the same time, it also has some limitations. In our approach, the agent was trained on a limited number of 3D block shapes, which could be enhanced and extended to objects found in daily life such as bottles, cups, balls, etc. By doing so and redesigning the whole approach structure on ROS/ROS2, we intend to perform testing on a real UR5 robotic arm using daily life objects, too. Efficient management requirements due to the feature map concatenation factor of DenseNet-121 can also be seen as a kind of limitation. Another limitation can be seen in the factor of our approach only involving a sequential combination of robotic manipulations, for which, in the future, the number of actions can be increased and parallel robotic manipulation combinations can be brought in, along with other new robotic manipulations such as stacking, rolling, rotating, etc. By implementing these extensions, the system may become so large that the overestimation of future rewards will undoubtedly cause problems. Therefore, to resolve this issue, Double Q-learning [62] and Dueling Q-learning [63] variants can be explored in the future in order to increase the chances of efficiency and throughput.

## Figures and Tables

**Figure 1:**
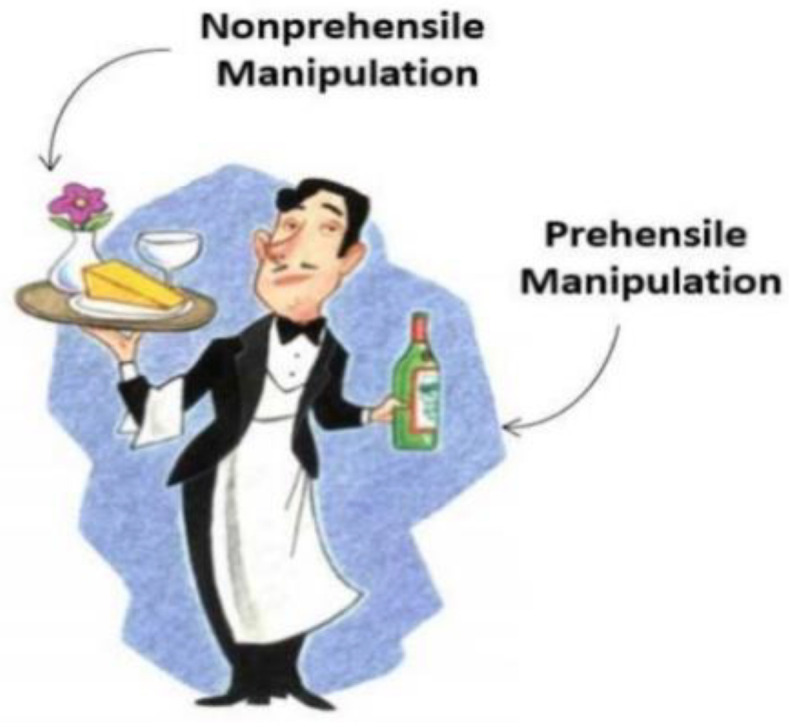
An example of prehensile and non-prehensile manipulations.

**Table 1:** Average Test Results (%).

Category	Success Rate	Grasping Success
Minimum Clutter	84%	96%
Medium Clutter	82%	95%
Maximum Clutter	74%	82%
Complicated Scenarios	65%	73%

## Data Availability

Data are available on request from the authors.
